# Control of Haemorrhage in Orthopaedic Trauma

**DOI:** 10.3390/jcm13144260

**Published:** 2024-07-22

**Authors:** Robert M. Kenyon, Jennifer L. Leighton

**Affiliations:** 1 Dalhousie University, Halifax, NS B3H 4R2, Canada; 2 Dalhousie University, Dartmouth General Hospital, Dartmouth, NS B3H 4R2, Canada

**Keywords:** orthopaedic, haemorrhage, trauma

## Abstract

This paper aims to outline current practices and examine promising new advancements in the modern management of haemorrhage in orthopaedic trauma. Many prehospital and perioperative haemorrhage control strategies and techniques have been available to clinicians for multiple decades, yet our understanding and utilisation of these practices continues to be refined and optimised. There is a particular focus in this article on issues related to resuscitation and coagulation in trauma. We examine the complex mechanisms that lead to coagulopathy in trauma patients as well as the transformative effect tranexamic acid has had in limiting blood loss. We also explore some emerging technologies such as endovascular interventions and clot-stabilising dressings and devices that are likely to have a significant impact going forward.

## 1. Introduction

Trauma remains the leading cause of mortality amongst 10–49-year-olds globally [1]. It is estimated that approximately 8% of all deaths occur as a result of trauma [2]. Haemorrhage is thought to be the leading cause of death in nearly half of these cases [3,4]. This picture has been relatively static over the past 30 years when compared with other causes of mortality [1]. Advances in both prehospital and hospital strategies to reduce blood loss have for the most part achieved only modest improvements in outcomes [4]. Prehospital strategies to reduce the morbidity and mortality associated with blood loss resulting from trauma include field interventions and prehospital resuscitation as well as the promotion of cultural and societal changes that aim to prevent or reduce the severity of traumatic injuries. The greatest advancements in the management of major trauma cases in the past two decades are likely attributable to improvements in resuscitation, transfusion and perhaps in particular the development of practices around the use of tranexamic acid [1,2,3,4,5]. Perioperative strategies and techniques to further reduce blood loss in major trauma patients continue to emerge. It is clear that to continue to advance our management of severely injured trauma, a systematic and wholistic approach towards reducing the morbidity associated with blood loss is essential. This review article attempts to outline the various strategies that are being implemented to reduce blood loss and also mitigate the effects of blood loss in trauma patients.

## 2. Injury Prevention

The Global Burden of Diseases, Injuries, and Risk Factors (GBD) Study routinely publishes figures estimating the trends in morbidity and mortality for all causes of trauma globally [6]. During the period from 1990 to 2017, there has been a greater than 40% increase in global population. The total number of deaths per year due to trauma has increased from 4,260,493 to 4,484,722 during this time; however, the age-standardised death rate related to all trauma has reduced from 1079 per 100,000 to 738 per 100.00 [6]. This gives credence to the assertion that a large proportion of trauma-related deaths by and large may be avoidable. The improvements are likely attributable to a combination of overall improvements in healthcare as well as road traffic safety initiatives and injury prevention programmes in wider society.

Arguably, prevention is the most important prehospital strategy in reducing blood loss and mortality related to trauma. Road traffic injuries remain the leading cause of trauma-related deaths both for males and females [6]. The implementation of road safety strategies as well as technological improvements in vehicle safety systems particularly pertaining to braking and behaviour at impact has been shown to have a significant effect on trauma-related morbidity and mortality [7]. Prophylactic strategies that aim to decrease the severity of injuries sustained during road traffic accidents have a significant role to play in reducing the burden of trauma and the likelihood of catastrophic blood loss. The combination of seat belts and airbags being employed in a motor vehicle collision is estimated to result in a reduction in occupant mortality by 67–80% [8,9]. Furthermore, the severity of injuries observed is significantly reduced [10,11].

Although not yet appreciable in practice at this time, one technological innovation that is likely to have a major impact on the incidence of road traffic-related injuries in the future in the developed world is that of vehicle automation or self-driving cars [12]. Combs et al. developed a model to hypothetically simulate how automated vehicles might potentially have performed in the context of 3386 real-life pedestrian fatality cases that they classified as potentially preventable [13]. They postulated a potential effectiveness ratio in avoiding the death of greater than 90% across all crash conditions when simulating a model of an automated vehicle employing a camera, LiDAR and radar sensors.

## 3. Prehospital Strategies

The Advanced Trauma Life Support system provides a framework for the initial assessment and emergent treatment of critically ill trauma patients [14]. Over the decades since its inception, it has become the gold standard for emergency trauma treatment and has been adopted in all settings from field use to use in emergency departments. Many of the advancements in emergent trauma care are conceived and implemented first in a military setting before being borne out into wider civilian society. The traditional structure of the ATLS system prioritises ABC (airway/breathing/circulation). Observations from battlefield scenarios have led to a relatively recent paradigm shift amongst many practitioners towards the need to greatly prioritise haemorrhage control in certain situations. For example, since 2006, the Battlefield Advanced Trauma Life Support used by the British armed forces has promoted the revised system of <C>ABC, with <C> denoting a priority towards “catastrophic haemorrhage” over the airway [15]. Although not formalised or officially practised in a civilian setting, there is some evidence to show that this way of thinking is being adopted to some degree in many major trauma centres [16].

Historically, the introduction of certain relatively simple interventions in the field has at times resulted in dramatic effects on the degree of blood loss amongst trauma patients. This is no better exemplified than with the advent of the Thomas splint. Stabilising a femur fracture under traction leads to a reduction in blood loss, reduction in pain and avoidance of conditions that may promote thromboembolism [17,18]. The adoption of the splint in the management of those in the British war wounded during World War I brought the mortality associated with femur fractures to 15.9%, down from an estimated 80% prior to its introduction [18]. Despite its inception in 1865, it remains relevant and fundamentally unchanged to this day. It continues to play an essential role in the modern emergent management of trauma, both in military and civilian contexts [19]. The timing of the splint application is important. Hoppe et al. demonstrated a statistically significant reduction in transfusion rate, as well as a reduction in pulmonary embolism rates amongst patients treated with a Thomas splint in the prehospital setting versus those treated with a Thomas splint after initial radiological investigation [17].

Unstable pelvic fractures pose a significant risk of major blood loss and mortality [20]. An estimated one in three polytrauma patients globally presenting with a pelvic fracture will die [21]. Mortality rates amongst patients with unstable pelvic fractures can be as high as 42% [22]. The application of pelvic circumferential binders has been shown to have a significant effect on haemodynamic stability in these patients as a result of a reduction in the pelvic volume, the stabilisation of the fracture and an increase in the intrapelvic pressure [23,24]. The use of pelvic circumferential compression devices is not entirely benign, however, and carries a risk of skin necrosis which appears to be duration-dependent [25]. It remains a topic that requires further study to definitively establish the risk–benefit ratio [26]. There has been a recent exploration into a potential change in practice away from the routine prehospital application of pelvic binders in all trauma patients and towards selective use in those patients with a suggestive mechanism of injury and evidence of haemodynamic instability [27,28].

Field application of tourniquets in a military context is a practice that has been shown definitively to be associated with improved survival likelihood [29,30]. A recent meta-analysis demonstrated a significant reduction in mortality amongst patients with vascular trauma when a tourniquet was applied in the prehospital setting (odds ratio of 0.48) [31]. No corresponding increased risk of amputation or compartment syndrome was observed. Once applied, a tourniquet might typically be left in situ until definitive haemostasis can be achieved surgically [29,30]. Case reports have illustrated severe complications arising as a result of tourniquets remaining in situ for more than 2 h; therefore, prompt action is essential [32]. Recent observations from the conflict in Ukraine have placed greater emphasis on the importance of a tourniquet conversion at the earliest possible juncture [33]. This principle is readily applicable in the civilian context. Tourniquet conversion refers to an attempt at tourniquet removal in exchange for a haemostatic dressing, whilst maintaining haemostasis.

Promising findings from military studies have emerged showing the potential benefits of prehospital initiations of blood transfusions. A retrospective cohort study performed by the US military demonstrated a significant reduction in 24 h and 30-day mortality rates following the prehospital transfusion of blood products [34]. Much focus has been placed on determining whether these findings can be extrapolated to a civilian context. The resuscitation with pre-hospital blood products (RePHILL) study was a multicentre randomised controlled study published in *The Lancet* in 2022. The results from the trial did not show that the prehospital transfusion of blood products (packed red blood cells and lyophilised plasma) was superior to resuscitation with a crystalloid solution [35]. Furthermore, the findings from a recent metanalysis performed by Schoenfeld support this stance. With data pooled from three randomised controlled trials, the authors found no statistically significant benefit in the 1-month mortality following the adoption of a civilian prehospital transfusion programme [36].

## 4. Tranexamic Acid

Tranexamic acid is a synthetic analogue of the amino acid lysine that acts as an antifibrinolytic, principally through the prevention of plasminogen activation. The Crash 2 trial was a seminal study that has been instrumental in establishing the practice of routine tranexamic acid administration amongst trauma patients. This large (20,211 patients) multicentre randomised placebo-controlled trial was originally published in *The Lancet* in 2010 [37]. The authors found a statistically significant reduction in the 28-day all-cause mortality from 16.0% to 14.5% and death due to bleeding from 5.7% to 4.9%. Crucially, the study only demonstrated a reduction in the risk of death due to bleeding if tranexamic acid was administered within 3 h of injury. The dose of tranexamic used in the study was a loading dose of 1 g over 10 min followed by an infusion of 1 g over 8 h, and this has been widely adopted in subsequent studies and clinical practice. The dramatic impact observed with this drug belies its inexpensiveness and widespread availability globally. The CRASH II trial also served to highlight the significant cost-effectiveness of this intervention, demonstrating a cost of USD 64 dollars per life year saved in a developed trauma network setting [38].

Critiques of the CRASH II trial have focused on the relative reliance of the study on data collected from countries that have less advanced trauma care or that have yet to establish structured integrated regionwide trauma systems. Detractors have suggested that this may have led to an overstating of the potential benefit of tranexamic acid, which may not necessarily be accurate when applied to a more developed trauma system [39,40].

Prehospital administration of tranexamic acid has been a mainstay in many resuscitation protocols used by military organisations around the world for some time [41]. Subsequent to the CRASH II trial, the American armed forces published the Military Application of Tranexamic Acid in Trauma Emergency Resuscitations (MATTERs) trial, a retrospective review of the outcomes of tranexamic acid administration in the treatment of combat-related injuries [42]. Patients included in the tranexamic acid group had received a 1 g IV bolus as the standard, with repeated doses administered at the discretion of the treating physician. They observed significantly lower mortality rates in those who had received tranexamic acid despite the tranexamic acid group having worse injury severity scores overall. The effect was most pronounced amongst a cohort of patients who received massive transfusions (>10 units within 24 h). The greatest benefits of tranexamic acid it seems are observed in those most severely injured. Other authors have found a reduced rate of multiorgan failure in severely shocked trauma patients undergoing tranexamic acid treatment [43].

Pre-hospital Antifibrinolytics for Traumatic Coagulopathy and Hemorrhage (PATCH-Trauma) trial was published in *The New England Journal of Medicine* in 2023 [39]. This was a randomised placebo-controlled trial examining the effect of the prehospital administration of tranexamic acid across multiple centres in Australia, New Zealand and Germany. Patients in the tranexamic acid group received a 1 g IV bolus followed by a further 1 g infusion in 1 L of saline over the course of 8 h. The study demonstrated a reduction in early mortality as a result of tranexamic acid administration, similar to that seen in CRASH II, with an odds ratio of 0.69. The study, however, showed no difference in the percentage of patients surviving with a favourable functional outcome at 6 months between the tranexamic acid and the placebo groups. Neither CRASH II nor PATCH studies demonstrated any increased risk of thromboembolism as a result of tranexamic acid administration [38,39].

An intraoperatively topical administration of tranexamic acid has been shown to be as effective as alternative routes of administration [44]. Currently, dressings that utilise the properties of tranexamic acid are not widely in use in civilian settings. CounterFlow-Guaze (Mitacs, Vancouver, BC, Canada), a relatively recent innovation, is a topical dressing that contains tranexamic acid as well as a microparticle dispersion system that has the potential to deliver pharmacological agents deeper into wounds. It has been developed for use in the military setting in particular and, so far, has demonstrated promising results in animal models [45]. Other proprietary dressings employing haemostatic agents that are currently in use in trauma care include QuikClot Combat Gauze (QCG) (Z-MEDICA, Wallingford, CT, USA) that contains kaolin (a factor XII activator) and chitosan-containing dressings such as HemCon and ChitoGauze (HemCon Medical, Tigard, OR, USA) or Celox Gauze (Celox Medical, Crewe, UK) [46,47]. Chitosan is a positively charged polysaccharide that promotes red blood cell and platelet aggregation [48]. XSTAT (REVMEDX, Wilsonville, OR, USA) delivers chitosan through an expanding cellulose sponge that is inserted internally with a syringe apparatus [47]. ResQFoam (Arsenal Medical, Waltham, MA, USA) also employs a syringe apparatus to deliver two liquid precursors within a cavity that react to form an expanding hydrophobic foam. ResQFoam is more typically indicated for non-compressible haemorrhage such as that encountered following intraabdominal trauma [49]. The effect of each of these agents is achieved both through pharmacological action and also through direct pressure and tamponade as a result of their specific delivery mechanism and physical properties.

## 5. Volume Replacement and Transfusion

The goal of fluid resuscitation in trauma is primarily to replace lost blood volume in order to maintain effective stroke volume and cardiac output, ultimately ensuring adequate tissue oxygenation and perfusion. At the same time, fluid strategies must be effective in controlling bleeding and maintaining appropriate content, concentration and temperature of the blood within the circulating volume. Overly aggressive fluid resuscitation practices have been shown to result in higher rates of mortality in certain contexts [50]. Damage control resuscitation is a strategy that employs philosophies of restricted volume replacement and permissive hypotension [30]. In conjunction with haemostatic resuscitation and damage control surgery, this strategy aims to avoid or mitigate the lethal triad of coagulopathy, acidosis and hypothermia [51]. There is a large volume of evidence from retrospective studies extolling the potential benefits of DCR; however, there is relatively little RCT evidence, and so it remains a topic of much debate [30,52].

Permissive hypotension is the practice of deliberately maintaining systolic blood pressure below the normal physiologic range during a critical phase in the early stages of treatment of the haemorrhagic trauma patient, with the aim of promoting the formation of a more robust clot and therefore reducing overall blood loss. The ‘permissive’ element refers to the controlled calculated exposure of the patient to the detrimental effects of reduced tissue perfusion, in particular, the potential exacerbation of brain injury. The concept appears to have been applied initially to the management of ruptured abdominal aortic aneurysms [53]. Much of our early understanding of the mechanisms and potential benefits of permissive hypotension are extrapolated from studies involving animal models. The relevance and transferability of these results may be questionable. Even within animal models, the observed benefits of hypotension can be dramatically different or even reversed depending on the site of the haemorrhage [54]. The optimum use of permissive hypotension in trauma patients remains somewhat elusive and controversial and is likely highly specific to the type of injury. The adoption of broad guidelines therefore is fraught with difficulty. Typically, in a trauma patient, a target systolic blood pressure of 80–90 mmHg may be employed initially until the control of major haemorrhage has been achieved [30]. NICE guidelines recommend that in the treatment of patients with haemorrhagic shock in which traumatic brain injury is thought to be the dominant condition, a less restrictive volume resuscitation approach is more appropriate [55].

The use of balanced crystalloids (e.g., Ringer’s lactate) compared with normal saline has been shown to result in reduced mortality from all causes amongst critically ill ICU patients [56]. In the context of traumatic brain injury, however, the use of Ringer’s lactate has been shown to increase mortality compared to normal saline [57]. The mechanism for this is not entirely clear; however, the relative hyperosmolality of normal saline compared to Ringer’s lactate is believed to result in a greater reduction in cerebral oedema [57].

Transfusion in trauma patients remains a challenging area for clinicians despite the advancements in our understanding and appreciation of blood products and their physiological effects. Determining the need for massive transfusion in a trauma patient based on traditional investigations and clinical judgement alone has been shown to be unreliable [58]. ATLS offers a grading system for estimating the degree of blood loss based on a number of clinical parameters [14]. This has been shown to be a useful clinical tool to aid rapid decision-making during resuscitation [59]. There are, however, many studies illustrating the limitations of its accuracy [60,61]. Transfusion protocols in trauma differ across the world and even differ between centres within the same hospital networks. For the three main products transfused (plasma; platelets; and red blood cells), typically a ratio of 1:1:1 or 1:1:2 is used [62]. Findings from the PRospective Observational Multicenter Major Trauma Transfusion (PROMMTT) study demonstrated that transfusion ratios of plasma to red blood cells and platelets to red blood cells that were ≥1:1 resulted in a more favourable outcome than those that were <1:2. This was only reflected in the 6 h mortality rates. Amongst survivors at 24 h and 30 days, transfusion ratios did not appear to have any association with mortality risk [63]. PROPPR was a multicentre randomised controlled trial comparing different ratios of the transfusion of plasma, platelets and red blood cells in major trauma patients [64]. Conversely to PROMMTT, the authors of PROPPR found that a ratio of 1:1:1 (plasma, platelets, red blood cells) achieved a greater degree of haemostasis and fewer deaths due to exsanguination within 24 h when compared to a ratio of 1:1:2. There was no difference, however, in the overall mortality rates at 24 h or 30 days between the two groups. The two studies differed fundamentally in design in that PROMMTT looked at outcomes when the transfusion ratio was closer to 1:1:1 or closer to 1:1:2 while PROPPR randomised patients to a fixed regimen of 1:1:1 or 1:1:2.

Repeated assessments of both haemoglobin and haematocrit are a mainstay investigation in the ongoing assessment of patient response to resuscitation [65]. Change in haematocrit during resuscitation is a highly reliable predictor of the persistence of bleeding [66]. The absence of a significant drop in haematocrit, however, should not be used to rule out major haemorrhage [65]. An analysis of a subset of the Transfusion Requirements in Critical Care (TRICCs) trial comparing trauma patients randomised either to a restrictive transfusion protocol (transfusing if haemoglobin <70 g/L) or a liberal protocol (transfusing if haemoglobin < 100 g/L) demonstrated that a restrictive protocol was safe [67]. A target haemoglobin of 70 g/L to 90 g/L is typically recommended [30].

It is recommended that pharmacological thromboprophylaxis be commenced within 24 h of achieving control of the bleeding [30]. LMWH has been proposed as the optimal thromboprophylactic agent in the context of multiple orthopaedic injuries [68]. The incidence of deep vein thrombosis amongst trauma patients not receiving any prophylaxis against thromboembolism was determined to be as high as 58% [69].

## 6. Coagulopathy

To understand the pathophysiology and thus the morbidity and mortality associated with major traumatic blood loss, it is important to consider a number of physiological mechanisms. In response to the depletion of circulating blood volume, there is an increase in heart rate and contractility as well as an increase in peripheral vasoconstriction in order to maintain tissue perfusion of the vital organs. Insufficient oxygenation of tissues results in a relative shift from aerobic metabolism to anaerobic metabolism. The lactic acid byproducts of this process drive a progressively worsening metabolic acidosis. Further and prolonged lack of adequate tissue perfusion and hypoxaemia can lead to local organ dysfunction. In addition to the deleterious effects of insufficient circulating volume and reduced tissue perfusion, physiological responses to trauma also lead to a systemic coagulopathic state. The mechanisms that drive these processes are multifactorial. The combination of metabolic acidosis, a depletion of circulating clotting factors lost to haemorrhage and hypothermia may ultimately result in coagulation cascade dysfunction. This phenomenon is exacerbated by, although not dependent on, a further reduction in the concentration of vital clotting factors that can occur as a result of the dilutional effect of volume replacement during resuscitation. Thus, it can be considered that there are two distinct processes that contribute in tandem to produce this overall clinical picture: acute coagulopathy of trauma and resuscitation-induced coagulopathy [70].

The presentations and complex mechanisms that cause the coagulopathic states that are observed in major trauma patients have been described under a number of different titles. These include Trauma-Induced Coagulopathy (TIC), Acute Traumatic Coagulopathy, Acute Coagulopathy of Trauma Shock (ACoTS) and Early Coagulopathy of Trauma. There is also considerable overlap with the condition of Disseminated Intravascular Coagulation (DIC) in the trauma patient. Our understanding of these entities continues to evolve and so does the nomenclature used in the published literature. ACoTS is a concept that was introduced in 2007 [71]. Initially, it was hypothesised that this process is activated primarily by tissue hypoperfusion and modulated through the protein C pathway. It has been suggested that this process may be independent of injury severity [71]. The results of other research have seemingly downplayed the significance of protein C activation in this process and suggest other pathways leading to systemic thrombin generation in the early stages of trauma [72]. Regardless, findings have consistently shown very poor prognosis in major trauma patients who develop coagulopathy. The presence of coagulopathy in a trauma patient confers a fourfold increased risk of mortality, with some studies reporting mortality rates nearly as high as 50% [73,74].

The monitoring of coagulation should begin immediately upon presentation of the traumatised patient [30]. Conventional coagulation tests such as Prothrombin Time (PT), International Normalised Ratio (INR) and Activated Partial Thromboplastin Time (aPTT) have significant limitations in the acute assessment of major trauma patients. These laboratory investigations are performed on centrifuged blood samples rather than whole blood samples resulting in an inherent delay in determining a result. One study has demonstrated a median turnaround time of 88 min for conventional coagulation testing compared with less than 5 min for some point-of-care tests [75]. In addition, the results of conventional tests do not necessarily convey all the information required to assess and treat coagulopathy in trauma [74].

Viscoelastic coagulation testing has an important role in the management of trauma patients and the mitigation of coagulopathy. This includes investigations such as Thromboelastography (TEG) and Rotational Thromboelastometry (ROTEM). Compared with conventional testing, the use of ROTEM in trauma patients has been shown to be associated with a reduced transfusion requirement and a reduced risk of mortality [76]. Both tests are point-of-care tests that measure the change in viscoelastic properties of a citrated or heparinised sample of blood as a clot is formed. In this way, the test rapidly generates a coagulation profile for the sample, providing detailed information about clot initiation, progression, stabilisation and dissolution [77]. Both tests are similar in concept and can be used to obtain similar, but not entirely equivalent, information profiles [74,77,78]. They each involve a specific apparatus used both to agitate the fluid and to detect changes in its properties. This is coupled with the addition of certain reagents specific to the clinical scenario and the coagulation profile desired. TEG is more typically found in widespread use in North America while ROTEM has gained traction throughout Europe [74]. Algorithms have been developed based on the profiles produced for use in the resuscitation of trauma patients to ensure a tailored administration of fibrinogen supplementation, fresh frozen plasma, platelets and tranexamic acid to accurately meet a patient’s requirements [74,78,79]. TEG and ROTEM may also be used to detect the presence of certain anticoagulants which may assist in the resuscitation of patients in which the background medical history is unknown [74]. Although viscoelastic tests may in some cases be able to distinguish between different classes of anticoagulant, such as Xa inhibition versus direct thrombin inhibition, they cannot necessarily determine the specific therapeutic agent present [80].

## 7. Perioperative and Intraoperative Strategies

Emergent preperitoneal pelvic packing is a relatively recent technique that has gained widespread adoption [81]. A number of variations of the preperitoneal pelvic packing process have been described [82,83,84]. Typically, a midline incision might be performed, although an approach through a Pfannenstiel incision may alternatively be used. If the patient is also undergoing a laparotomy, the pelvic packing incision should be kept separate. The tamponade effect of the pelvic packs inserted is dependent on the mechanical stability provided by the application of an external fixator or a pelvic binder [84]. The role of the pelvic external fixator is to maintain mechanical stability, either as a temporary measure whilst haemodynamic stability is established or as a definitive treatment of an unstable pelvic fracture. Pelvic binders and external fixators or clamps have been shown to achieve comparable degrees of mechanical stability and efficacy in haemorrhage control in some instances [85,86]. The advantage of the external fixator or clamp over the pelvic binder is the mitigation of soft tissue or skin compromise as a result of prolonged external compression as well as facilitating greater access for definitive surgical management.

With haemodynamically unstable pelvic fractures, typically the source of bleeding is predominately venous, resulting from disruption to the presacral and paravesical venous plexus [84]. It is estimated that 10–20% of bleeding is attributable to arterial sources [87]. If bleeding persists despite the establishment of mechanical stability, then significant arterial bleeding is more likely to be present [88]. Pelvic angioembolisation is a minimally invasive endovascular technique that can be particularly effective in controlling haemorrhage when arterial bleeding predominates [81,84]. There is strong evidence to support the emergent use of angiography/angioembolisation in pelvic fracture patients exhibiting haemodynamic instability or radiographic evidence of arterial contrast extravasation [81,84].

Studies to date that have attempted to draw a direct comparison between pelvic packing and angioembolisation have not conclusively been able to demonstrate a definite advantage of one treatment modality over the other with regard to the mortality rate [89,90,91,92,93]. This is likely a reflection of the heterogeneity of these patients and their pathology as well as the propensity for the overlapping of treatments. A recent systematic review and meta-analysis mainly featuring data from retrospective and prospective cohort studies found that 27% of those patients initially treated with pelvic packing ultimately went on to have embolisation as well due to failure to control the bleeding [90]. It appears that angiographic embolisation may be employed as a first-line treatment in a haemodynamically stable patient, demonstrating radiological evidence of arterial bleeding; however, in haemodynamically unstable patients’ pelvic packing, stabilisation and adjunct endovascular treatments may be required [81,84,88,93]. Subsequent or repeated angioembolisation may also be indicated in certain instances [81,84].

Resuscitative Endovascular Balloon Occlusion of the Aorta (REBOA) is an emerging technology that some believe has the potential to revolutionise haemorrhage control in certain cases of severe trauma [94,95]. The technique involves the inflation of a balloon within the aorta to limit blood flow distally. This may be performed in the thoracic aorta for higher injuries or the abdominal aorta as in the case of a massive haemorrhage from a pelvic fracture. This is performed as a stopgap, limiting blood loss until definitive haemorrhage control can be achieved through embolisation or surgical means [96]. A meta-analysis of data from cohort and observational studies has shown promising outcomes [95,96]. More recently Jansen et al. published a randomised controlled trial in *JAMA* in 2023 which demonstrated contrasting results [97]. The study compared REBOA and standardised care to standardised care alone in 90 patients who were deemed to be potentially amenable to the treatment. They found that there was no demonstrable benefit from REBOA in this cohort and indeed actually observed a higher 90-day mortality rate amongst the REBOA group (54% versus 42%).

## 8. Trauma Systems

Many acute hospitals around the world are not equipped to adequately deal with the complexities of the multiply injured patient. This problem can be further compounded when multiple complex trauma patients present simultaneously. Many hospital networks have established trauma systems, which exist in part to ensure that such patients are transferred to a centre which is best adapted to deal with them. This may occur as a transfer between emergency departments or strategic redirection of ambulance services from their typical protocol to ensure direct presentation at the most appropriate centre. The argument against the direct transfer of severely injured patients to a major trauma centre with the bypassing of local emergency services is that the additional transit time may potentially result in adverse outcomes. This is a complex issue as there are multiple economic and social factors that contribute to this, and observations from one jurisdiction may not necessarily be transferrable to another. At present, there does not appear to be a definitive consensus in the literature with regard to the bypassing of local services [98]. However, when the so-called “hub and spoke” model for trauma care has been applied with effective integration between emergency medical services and hospital-based services, significant improvements in patient outcomes and efficiency of services have been observed [99].

Regardless of the particular models and protocols adopted within a trauma network, the establishment of trauma systems in general has been shown to result in a 15% reduction in mortality amongst trauma patients [100]. An inter-hospital transfer of trauma patients does not appear to have an impact on mortality [101]. There is compelling evidence to support the centralisation and specialisation of trauma care [99,102,103]. Perhaps, unsurprisingly, the benefits from these strategies are most pronounced for those most severely injured [103].

## 9. Conclusions

Limiting blood loss and mitigating the morbidity associated with the sequelae of haemorrhage is critical in the management of major trauma patients. There are many strategies that can be employed to directly control bleeding and to safely and effectively resuscitate the critically injured patient. Emerging technologies offer potential opportunities to develop treatments for patients with traumatic injuries that might previously have been considered un-survivable. It is clear that a progressive approach to the management of trauma goes beyond just the techniques employed in the prehospital and perioperative setting and instead involves a comprehensive and systematic process encompassing all aspects of trauma care from prevention to resource coordination.
